# An incidental case of biliary fascioliasis with subtle clinical findings: US and MRCP findings

**DOI:** 10.2478/raon-2013-0021

**Published:** 2013-05-21

**Authors:** Hakan Önder, Faysal Ekici, Emin Adin, Suzan Kuday, Hatice Gümüş, Aslan Bilici

**Affiliations:** Department of Radiology, Dicle University Medical Faculty, Diyarbakır, Turkey

**Keywords:** fascioliasis, ultrasonography, magnetic resonance cholangiopancreatography

## Abstract

**Background:**

Fascioliasis is a disease caused by the trematode *Fasciola hepatica.* Cholangitis is a common clinical manifestation. Although fascioliasis may show various radiological and clinical features, cases without biliary dilatation are rare.

**Case report:**

We present unique ultrasound (US) and magnetic resonance cholangiopancreatography (MRCP) findings of a biliary fascioliasis case which doesn’t have biliary obstruction or cholestasis. Radiologically, curvilinear parasites compatible with juvenile and mature *Fasciola hepatica* within the gallbladder and common bile duct were found. The parasites appear as bright echogenic structures with no acoustic shadow on US and hypo-intense curvilinear lesions on T2 weighted MRCP images.

**Conclusions:**

Imaging studies may significantly contribute to the diagnosis of patients with subtle clinical and laboratory findings, particularly in endemic regions.

## Introduction

Hepatobiliary fascioliasis is caused by the trematode *Fasciola hepatica (F. hepatica)*. The worldwide increase in the diagnosis of fascioliasis is related to the increased availability and use of ultrasonography, awareness of the important role of imaging in diagnosis, and the recent development of specific serologic tests. The parasite is common in sheep, goat and cattle. It is transmitted to humans via contaminated water or green vegetables such as watercress. The disease is endemic in some Middle and Far East countries and in some parts of Central and South America.^1^,^2^

Human fascioliasis mainly involves the hepatobiliary system. It has two different phases: hepatic (acute), and biliary (chronic) phase. The hepatic phase of the disease occurs when immature parasites pass into the liver through its capsule. The parasites migrate through the liver parenchyma to the biliary system. Biliary phase of the disease occurs in the presence of parasites in the biliary system.^2^,^3^

Typical radiological findings of biliary fascioliasis have been reported previously.^4^,^5^ Herein we present a case of biliary fascioliasis with no biliary obstruction and cholestasis; unique MRCP and US imaging findings.

## Case report

33-year-old female patient from the south-east part of Turkey has been referred to the department of gastroenterology with a stomach ache complaint. Laboratory results and physical examination were normal, except tenderness in the right upper quadrant and high erythrocyte sedimentation ratio.

MRCP revealed heterogeneous intensities within the gallbladder along with mild dilatation of common bile duct and intra-ductal linear hypo-intense lesion which was reached at 2 cm length on T2 weighted series (Figure 1A, B). Heterogeneous lesions at right liver lobe with maximum size of 1.5 × 1cm were also noted. *F. hepatica* was suspected since our region was endemic for that parasite. Three days after MRCP imaging, the US examination of the patient had depicted multiple mature *F. hepatica* parasites in the gallbladder and main bile ducts lumen with approximately 2 cm mean size (Figure 2A,B). Stool sample was (+) for *F. hepatica and Helicobacter pylori*. In ELISA testing, the result was read photometrically at 450 nm (Tecan Sunrisemicro ELISA). The absorbance value of the patient was 18 DRG Units=DU/ml. The excretory/ secretory antigens was used for immuno-diagnosis of fascioliasis in the kit (values greater than 11.0 DRG Units=DU/ml are interpreted as seropositive, cut-off value 10).

The patient received 10 mg/kg of triclabendazole therapy per day. Six weeks after initial treatment, erythrocyte sedimentation rate value returned to normal and parasites were not seen any more with the US and MRCP investigation.

## Discussion

*F. hepatica* is a zoonosis which can rarely infect people who have an oral contact with water or water plants contaminated by the larvae. Two different phases of fascioliasis with distinct signs and symptoms have been described. Hepatic (acute) phase is characterized by right upper quadrant pain, hepatomegaly, intermittent fever, urticaria and marked eosinophilia. In biliary (chronic) phase, patients have dyspeptic symptoms and intermittent right upper quadrant pain with or without cholestasis. Overlaps between both phases may occur.^6^–^8^

The diagnosis of fascioliasis is based on clinical symptoms, stool examination, serological and radiological studies the egg production rate of *F. hepatica* is low and. Therefore, stool examination is not very sensitive. Presently, serological studies are the main diagnostic tool and the most common serological method is ELISA which detects antibodies to the excretory-secretory antigen products from *F. hepatica*.^9^,^10^ Our patient had both positive serological tests and stool examination.

The presence of parasites in the biliary system in the chronic phase of the disease, is characterized by cholangitic fevers and dyspepsia caused by partial biliary obstruction. In this phase, US is able to demonstrate the floating parasites in the biliary system and accompanying oedema of gall-bladder or distal bile duct. Parasites usually appear as leaf-like or snail-like non-shadowing oval echogenic structures.^6^–^8^ In our case, intraluminal floating parasites were visible but concomitant biliary system oedema was not present.

In case series of five patients, Koç *et al.* found hypo-intense expansive filling defect due to *F. hepatica* on T2W images on MRCP of one particular patient.^11^ Linear hypo-intense appearance with no luminal expansion on MRCP was seen in images of our patient.

Parasites have variable length between 5 and 25 mm, but their characteristic size is 10 mm. In our case parasites were not at the same location on US and MRCP images. In our opinion it might be due to the movement of parasites during the time period between the examination dates.

Cholangitis is a common clinical manifestation of biliary fascioliasis. Gallbladder or common bile duct wall thickening along with mild intrahepatic biliary ductal dilatation frequently occur in these cases.^8^,^9^,^12^ Mild dilatation of common bile duct with no apparent oedema was found in our case.

In conclusion, fascioliasis should be a differential diagnosis when bile duct dilatation or intraluminal curvilinear structures are encountered on the US or MRCP images of patients with subtle clinical findings, particularly at endemic regions. Imaging findings should be supported by ELISA testing. Fascioliasis may show various radiological features and radiologists should be aware of this information.

## Figures and Tables

**FIGURE 1A:**
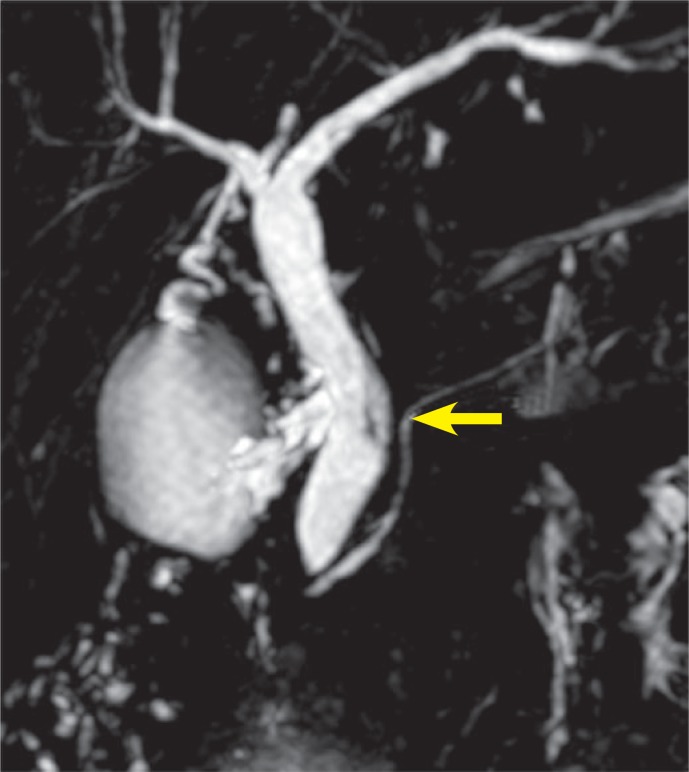
MRCP image shows mild dilation of the common bile duct and linear hypointense signal changes in the distal common bile duct *(arrow)*.

**FIGURE 1B:**
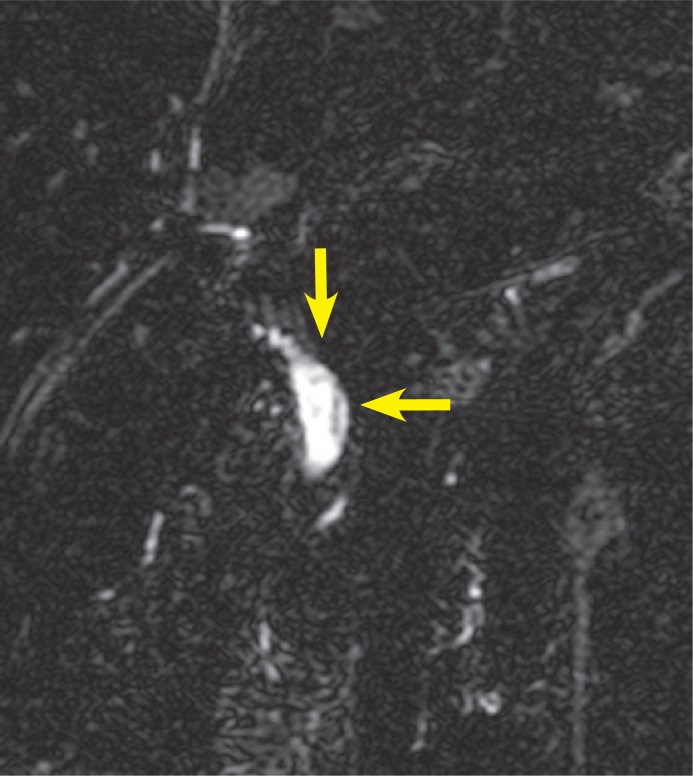
Thick-section (7 mm) MRCP image demonstrates the curvilinear parasite.

**FIGURE 2A:**
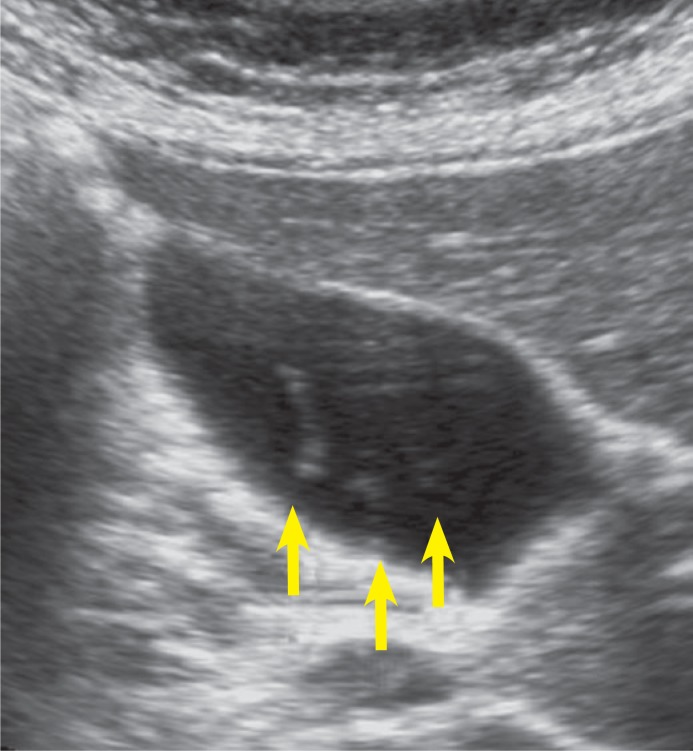
Transverse sonogram shows floating echoes (*arrows*) with no acoustic shadowing in the gallbladder of patient with fascioliasis.

**FIGURE 2B:**
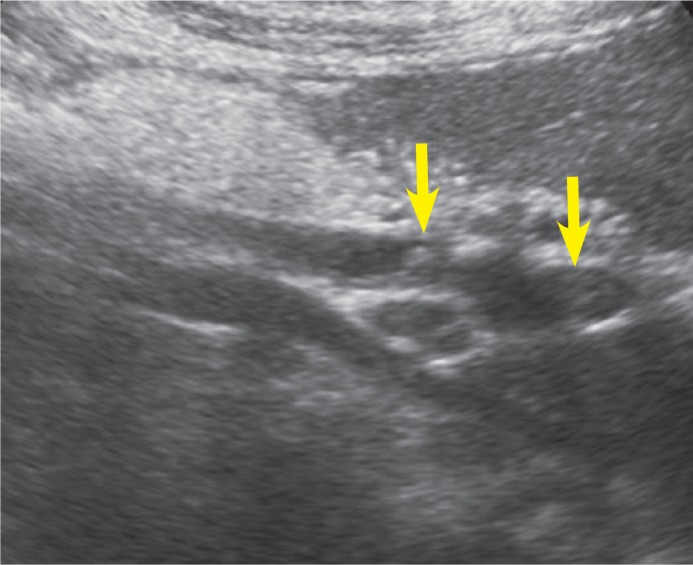
Sonogram of the common bile duct depicts curvilinear echogenic parasites *(arrow)*.

## References

[1] Arjona R, Riancho JA, Aguado JM, Salesa R, Gonzales-Macias J (1995). Fascioliasis in developed countries: a review of classic and aberrant forms of the disease. Medicine.

[2] Aksoy DY, Kerimoglu U, Oto A, Ergüven S, Arslan S, Unal S (2005). Infection with Fasciola hepatica. Clin Microbiol Infect.

[3] Pagola Serrano MA, Vega A, Ortega E, Gonzalez A (1987). Computed tomography of hepatic fascioliasis. J Comput Assist Tomogr.

[4] Yeşildağ A, Şenol A, Köroğlu M, Koçkar C, Oyar O, Işler M (2010). Hepatobiliary fascioliasis: a case with unusual radiological features. Diagn Interv Radiol.

[5] Yesildağ A, Yıldız H, Demirci M, Gören I, Isler M (2009). Biliary Fascioliasis:Sonographic appearance patterns. J Clin Ultrasound.

[6] Han JK, Choi BI, Cho JM, Chung KB, Han MC, Kim CW (1993). Radiological findings of human fascioliasis. Abdom Imaging.

[7] Kabaalioglu A, Cubuk M, Senol U, Cevikol C, Karaali K, Apaydın A (2000). Fascioliasis: US, CT, and MRI findings with new observations. Abdom Imaging.

[8] Richter J, Freise S, Mull R, Millan JC (1999). Fascioliasis: sonographic abnormalities of the biliary tract and evolution after treatment with triclabendazole. Trop Med Int Health.

[9] Kabaalioglu A, Apaydin A, Sindel T, Lüleci E (1999). Fascioliasis: US-guided gallbladder aspiration: a new diagnostic methods for biliary fascioliasis. Eur Radiol.

[10] Markel EK, John DT, Krotoski WA, Markel EK, John DT, Krotoski WA (1999). Immunodiagnostic techniques. Markel and Woge’s medical parasitology.

[11] Koç Z, Ulusan S, Tokmak N (2009). Hepatobiliary fascioliasis: imaging characteristics with a new finding. Diagn Interv Radiol.

[12] Ooms HWA, Puylaert JBCM, van der Werf SDJ (1995). Biliary fascioliasis: US and endoscopic retrograde cholangiopancreatography findings. Eur Radiol.

